# Influence of Post-Harvest Processing on Functional Properties of Coffee (*Coffea arabica* L.)

**DOI:** 10.3390/molecules28217386

**Published:** 2023-11-01

**Authors:** Michał Halagarda, Paweł Obrok

**Affiliations:** Department of Food Product Quality, Krakow University of Economics, Ul. Sienkiewicza 5, 30-033 Krakow, Poland

**Keywords:** coffee processing, antioxidants, polyphenols, caffeine, full washed, natural, anaerobic, washed–extended fermentation

## Abstract

Coffee is one of the most popular beverages worldwide, valued for its sensory properties as well as for its psychoactive effects that are associated with caffeine content. Nevertheless, coffee also contains antioxidant substances. Therefore, it can be considered a functional beverage. The aim of this study is to evaluate the influence of four selected post-harvest coffee fruit treatments (natural, full washed, washed–extended fermentation, and anaerobic) on the antioxidant and psychoactive properties of Arabica coffee. Additionally, the impact of coffee processing on the selected quality parameters was checked. For this purpose, results for caffeine content, total phenolic content (TPC), DPPH assay, pH, titratable acidity, and water content were determined. The results show that natural and anaerobic processing allow the highest caffeine concentration to be retained. The selection of the processing method does not have a significant influence on the TPC or antiradical activity of coffee. The identified differences concerning water content and pH along with lack of significant discrepancies in titratable acidity may have an influence on the sensory profile of coffee.

## 1. Introduction

Coffee is one of the most popular beverages worldwide. It is highly valued for its sensory properties as well as for its psychoactive effects that are associated with caffeine content. However, coffee is also a good source of antioxidant compounds, mainly phenolics, but also Maillard reaction products that are generated in the roasting process [1,2,3,4]. This makes coffee a functional beverage [5]. The roasting process itself has been thoroughly studied in terms of its impact on the antioxidant properties of coffee [2,6,7,8,9,10]. The impact of brewing time and method have also been verified [1,11]. However, post-harvest (pre-roasting) coffee bean preparation steps have an influence on their exact chemical composition [1,9,12] and thus may affect the content of compounds responsible for antioxidant activity and caffeine concentration.

The essence of coffee plant cultivation is extraction of its beans. To obtain them the fruit must be processed. Each of the layers covering the coffee bean when processed may affect the chemical composition of the bean itself. Therefore, coffee makers use different methods of coffee fruit treatment to achieve certain flavors. However, fruit processing not only has an impact on flavor precursors but may also influence the content of functional compounds. The fruits are processed immediately after harvesting to limit the occurrence of unwanted fermentation and reduce contamination. The most common methods include natural and full washed processing [13,14].

The dry method of coffee processing, also known as the natural method, is one of the oldest techniques for processing coffee cherries. In this method, fruits are spread in thin layers and dried in the sun. Depending on the specific region or place of cultivation, the drying stations may look different; some plantations will use the simplest brick terraces for this purpose, while others will use special beds that allow air to flow freely between the fruits, thanks to which drying takes place more evenly. The fruit is turned regularly to avoid mold, rotting, or fermentation. On larger plantations, mechanical drying devices are sometimes used to speed up the process [15]. However, this may have an influence on the coffee quality [16]. The sun-drying process itself takes about 3–4 weeks, until the cherries become hard to the touch, shrink, and take on a dark brown color [17]. When the fruit reaches a moisture content close to 11%, it is considered dry [18]. Then, to achieve higher quality, beans can be stored for some months in special silos. There they rest and the flavor of the beans matures fully [17]. This method ends when the skin and pulp are mechanically removed from the fruit, and the coffee beans are sorted, bagged, and exported to customers [19]. The dry method is used for approximately 90% of the Arabica coffee produced in Brazil, most of the coffee produced in Ethiopia, Haiti and Paraguay, and some Arabica produced in India and Ecuador. Almost all Robusta coffee is processed using this method [17,20,21]. The natural process is common primarily in places where there is no access to water. However, it is not practical in very rainy regions where the humidity is too high or where it often rains during the harvest months. Regardless of the variety and region of cultivation, the dry process primarily gives the coffee a fruity aroma and sweetness, as the drying process enhances the sugar profiles [21].

The full-washed coffee cherry processing method is by far the fastest, probably the most efficient, and therefore the most commonly used method. The first stage of the process is placing freshly picked cherries in a flotation tank filled with water, in which the ripe cherries sink and the unripe cherries—undesirable in harvesting—float to the surface, making it possible to remove them from further stages of processing [17,22]. The next step is to transfer the fruit to the depulper—a device that is responsible for splitting and squeezing the coffee cherry to separate the beans from the outer skin and pulp. After depulping, the coffee beans are still covered with a thin and sticky layer of mucus. Their resistance to pressure is due to the combination of sugars and pectins, and the best way to remove it is a fermentation process [13,15,17]. For this purpose, coffee beans are placed in fermentation tanks filled with water for 6 to 72 h [17,22,23]. During this time, thanks to the activity of enzymes, the pectins contained in the mucus are broken down. The duration of fermentation depends on many factors, including: altitude above sea level, ambient temperature, volume of coffee, and type of beans. The fermentation period has a significant impact on the flavor of the coffee, so knowing when to stop fermentation is a key factor in this process. If fermentation takes too long, undesirable flavors may occur. However, when properly carried out, washed coffee can acquire a characteristic, clean acidity [22]. Furthermore, the presence of bacteria and fungi that are specific for different areas and altitudes may affect the sensory profile of fermented coffee. The most common microorganisms that could be associated with coffee and its processing include: *Debaryomyces*, *Pichia*, *Candida*, *Saccharomyces kluyveri, S. Ceverisiae*, *Aspergillus*, *Penicillium*, *Fusarium, Trichoderma*, *Lactobacillus*, *Bacillus*, *Arthrobacter*, *Acinetobacter*, and *Klebsiella* [14]. After the fermentation process, the beans are washed again with clean water and left to dry in the sun. As with the dry method, beans can be dried on concrete terraces, tables, or beds. Depending on the prevailing weather conditions, the drying period may last up to 21 days, but is mostly completed between 2 and 15 days [15,22]. Already dried beans covered with parchment have a light beige color. To remove this thin layer, the beans are transferred to a dry mill where the parchment is rubbed off their surface. The final stage is the sorting and packaging of green coffee beans [15]. The taste and aroma of washed coffee can be described as a clean, light-bodied profile with pronounced acidity [24].

The washed–extended fermentation method is used when a given batch of coffee is harvested over several days. Each day, extracted beans are added to the fermentation tanks containing the previous days’ harvest. In this method, each subsequent batch of beans increases the pH level in the tank. This inhibits the growth of bacteria present in an acid environment, the activity of which may lead to excessive fermentation of the bed. Such a processing results in more sophisticated flavor profile of the coffee beans [25].

For some time now, in the world of specialty coffees, new coffees produced using new coffee cherry treatment techniques can be seen on the shelves. Experimental trials of new coffee processing methods began when Sasa Sestic won the 2015 World Barista Championship with carbon maceration coffee. Since then, many coffee producers, wanting to increase the cupping score of their coffees, have been trying their hand at producing perfect beans using innovative methods. Of these, anaerobic fermentation has attracted the most interest. The first stage is like that in the wet method. The fresh fruits are placed in a depulper, and then the separated beans are placed together with part of the pulp and outer skin in vacuum-sealed tanks equipped with a non-return valve to stop air from entering the tank. During the process, microorganisms begin to break down glucose molecules, resulting in the release of heat and carbon dioxide, which, being a heavier gas, displaces oxygen from the tank. In an anaerobic environment, bacteria naturally found in coffee cherries produce enzymes that break down sugars into less complex compounds such as organic acids or alcohols. Anaerobic fermentation allows for better control of the process by measuring the pH, sugar content, and temperature inside the tank. Controlling and prolonging the fermentation of coffee causes a change in its chemical composition, something which is also associated with changes in the flavor profile [23,26]. Coffees from this method are characterized by a silky, creamy texture and complex acidity [27].

Coffee studies considering post-harvest (pre-roasting) processing focus mainly on sensory characteristics, e.g., [14,26,28,29]. However, research concerning the impact of coffee fruit processing methods on the functional properties of coffee is scarce. Therefore, the aim of this study was to evaluate the influence of post-harvest coffee fruit treatments on the antioxidant and psychoactive properties of coffee. Furthermore, to the best of the authors’ knowledge, the manuscript presents the results of the first research directly comparing four different coffee fruit processing methods (natural, full washed, washed with extended fermentation, and anaerobic). Implementation of the processing on a coffee plantation allowed the quality of coffee cherries and the influence of the actual process conditions to be maintained, along with that of the site-specific microbiota to be reflected in the final characteristics of coffee beans.

## 2. Results and Discussion

The results of the study (Table 1) show that the water content was significantly higher in coffee beans from full-washed (2.68 ± 0.03 g/100 g) and anaerobic (2.63 ± 0.02 g/100 g) processing than in beans processed with the natural method (2.24 ± 0.03 g/100 g). These differences may be a consequence of the strong dehydration resulting from the sun drying used in natural processing. Other methods involve immersing coffee in water. The level of the retained water in natural processed coffee is consistent with the results obtained by Baggenstoss et al. [30] indicating that natural processed coffee after the roasting process contains 2.3 g/100 g of water.

The pH value was significantly higher in washed–extended fermentation coffees (5.08 ± 0.03) than in coffee beans processed using the anaerobic method (4.98 ± 0.02). Although both methods rely on fermentation, in the case of anaerobic coffees the process is carried out to a specific pH value of fermenting mass. Still, due to the continuous addition of successive portions of fresh beans to the fermentation tank, coffee beans from prolonged fermentation treatment tend to have a higher pH. This is connected with additions of extra amounts of sugars when dosing subsequent portions of beans. They are a product of the degradation of organic compounds and subsequently act as a nutrient medium for microorganisms to produce acids and alcohols [25]. The average titratable acidity of the tested coffees range from 18.5 to 18.83 with no significant differences. In view of this fact, it can be confirmed that the processing conditions influence the acidic profile of the coffee, which in turn has an influence on the sensory parameters of the beverage.

**Figure 1:**
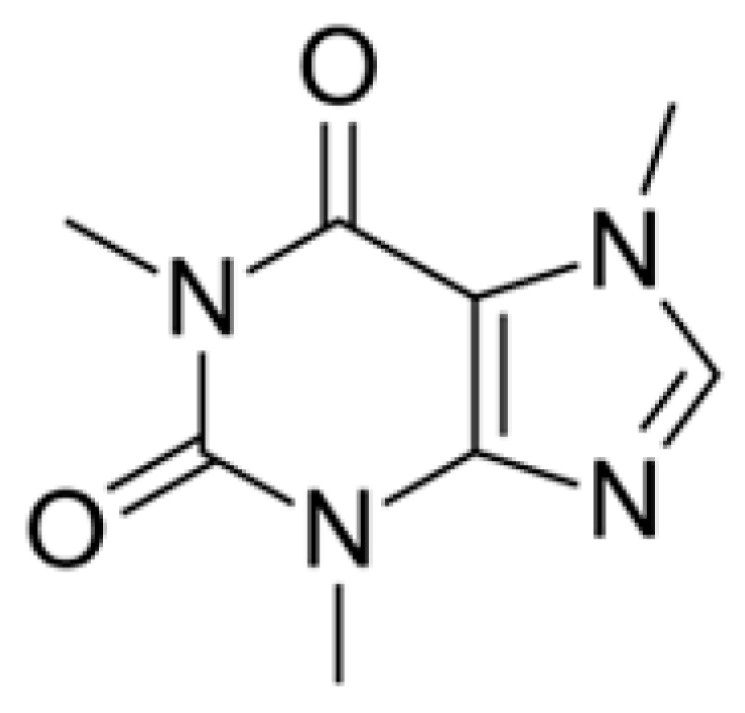
Chemical structure of caffeine.

The presence of caffeine, which has a centrally excitatory effect resulting from its structure (Figure 1), makes coffee a functional beverage. The more caffeine is in the bean, the more will diffuse into the beverage and the higher the stimulating effect for a coffee consumer. In this study, each of the coffees contained the amount of caffeine typical for the *C. arabica* (0.7–1.7 g/100) [31]. Lower values were noted by Eshetu et al. [32] for full- washed sun-dried Ethiopian Arabica (1.06–1.28 g/100 g, depending on variety). The results of this study indicated that the fruit-processing-influenced caffeine content in the roasted beans. The caffeine concentration in anaerobic coffees (1.758 ± 0.014 g/100 g) was significantly higher than in washed–extended fermentation coffees (1.672 ± 0.010 g/100 g) and full-washed coffees (1.666 ± 0.009 g/100 g). Moreover, coffee beans processed with the use of the natural method (1.758 ± 0.008 g/100 g) contained significantly more of this functional compound than full-washed coffees. This is in agreement with the findings of Guyot et al. [33], who detected small losses of caffeine (3%) as a result of the soaking phase in the wet process in comparison to the natural process. In contrast, Mintesnot and Dechassa [34] did not report any difference between caffeine content in coffees processed with the dry and wet method.

The functional properties of coffee are also connected with the content of antioxidant compounds, mostly polyphenols. The total phenolic content (TPC) ranges between 37.51 and 40.12 mg GAE/g. It seems to be typical for coffees from Indonesia, as those values are close to that determined by Jeszka-Skowron et al. [35]—38.5 mg GAE/g. At the same time, the determined TPC was higher than the values noted by Odžaković et al. [2] for Brazilian Arabica of three roasting degrees (23.66–32.78 mg GAE/g), as well as those for Chinese (36.17 mg GAE/g) and Thai (33.76 mg GAE/g) coffees studied by Cheong et al. [5] and was lower than the TPC determined by Bobková et al. [6] for light roasted coffees of Colombian, Indian, and Ethiopian origin (38.34–59.79 mg GAE/g) as well as by Cheong et al. [5] for Indonesian coffee (48.51 mg GAE/g).

The fruit processing itself did not have significant influence on TPC. Correspondingly, there were no significant differences in antiradical activity among the tested coffees. Nevertheless, Haile Bae and Kang [36] showed that, when considering light roasted coffees, wet processed coffee exhibits better antiradical activity against DPPH and higher TPC than the dry processed equivalent. However, surprisingly, in their research, there were no differences in DPPH inhibition for medium and dark roasted coffees, whereas discrepancies in TPC were noted for medium roasted beans.

The values of DPPH IC₅₀ obtained in this study seem to be typical and close to those measured by Vignoli, Bassoli, and Benassi [3] for light, medium, and dark roasted Arabica extract (16.11–24.92 μg mL^−1^).

## 3. Materials and Methods

### 3.1. Coffee Samples

Research samples of Arabica (*Coffea arabica* L.) S-795 cultivar beans were acquired from a plantation in the village of Beiposo, located in the Indonesian island of Flores. Coffee from Flores is of high quality thanks to the know-how of the local women who cultivate it. Only fully ripe cherries are harvested. Furthermore, they are grown in the fertile lands of the Bajawa Plateau, located between two volcanoes, at altitudes from 1300 up to 1600 m above sea level. Due to the favorable growing conditions and care for the quality of harvested fruit, the coffee from Beiposo is mild and has a balanced acidity and bitterness. Therefore, it is considered to be a specialty coffee [37].

The coffee cherries used for samples were processed using four different methods:

- Natural: coffee cherries were dried under the sun for 30 days.
- Full washed: coffee cherries were washed and then fermented for 24 h inside the tank with filled with water. The external temperature during fermentation was kept between 11 °C and 20 °C. The fermentation process was finished when the pH level reached 4.4.
- Washed–extended fermentation: Coffee cherries were washed and fermented 5 times for 24 h. Each fermentation process was followed by washing. The external temperature during fermentation was maintained between 12 and 18 °C.
- Anaerobic: The coffee cherries were fermented inside vacuum-sealed containers for 7 days, until the pH level reached 4.2.

The bean roasting process was performed using an SR3 Coffee Roaster (Coffed, Piła, Poland) equipped with temperature sensors placed in the roasted coffee and exhaust fumes. The process parameters were monitored using Artisan 2.4.6 software. The coffee beans were roasted to the same, light degree. For the purpose of the analyses the coffees were ground with the use of a Retsch GM 200 (Haan, Germany) mill.

### 3.2. Water

Water content was determined with the use of the oven-drying method according to the PN-A-76100:2009 Standard [38]. Samples of 5 g were dried at 103 ± 2 °C for 2 h. Afterwards they were cooled to room temperature in a desiccator and weighed. The procedure was repeated until a constant weight of dried sample was reached.

### 3.3. Titratable Acidity and pH

Titratable acidity and pH were measured according to the AOAC methodology [39]. The samples of coffee beans were milled. The samples of 2 g of coffee were homogenized with 100 mL water, kept in a water bath (60 °C) for 30 min, and cooled to room temperature. The pH values were measured using a SevenCompact digital pH meter (Mettler Toledo, Greifensee, Switzerland) equipped with an InLAb Expert Pro-ISM (Mettler Toledo, Greifensee, Switzerland) electrode. The titratable acidity was measured with the use of 0.1 mol L^−1^ NaOH and to pH 8.2.

### 3.4. Caffeine Content

Caffeine concentration was determined according to the methodology proposed by the ISO 20481:2008 Standard [40]. The sample of 1 g of milled coffee was mixed with 5 g of magnesium oxide and 200 mL of water, heated to 90 °C and kept at that temperature for 20 min. Then the solution was cooled down to room temperature and filled up with water to 250 mL. After filtration through a 0.22 µm PTFE filter, the sample was ready for HPLC analysis.

The HPLC analysis was performed with the use of a UltiMate 3000 RSLC (Thermo Fisher Scientific, Waltham, MA, USA) equipped with an Accucore XL C18 column (4.6 × 150 mm, 4 µm particle) and a DAD detector. The mobile phase (methanol in water 24% *v*/*v*) flow rate of 1.0 mL/min and isocratic elution were used. UV detection was performed at 272 nm.

### 3.5. Total Phenolic Content

Determination of the total phenolic content (TPC) was performed with the Folin-Ciocalteu method [3]. A sample of 0.1 mL of coffee solution (3 mg/mL) was diluted with deionized water to 7.5 mL. Subsequently, 0.3 mL of 0.9 M Folin-Ciocalteu reagent and 1 mL of 20% Na_2_CO_3_ solution were added, and the volume was filled up to 10 mL with deionized water. The solutions were kept at room temperature for one hour, and then the absorbance at 765 nm was measured with a Nanocolor UV/VIS II spectrophotometer (MACHEREY-NAGEL, Düren, Germany). Standard solutions of gallic acid were used to create the calibration curve. The results were therefore expressed in grams of gallic acid equivalents per 1 g of coffee.

### 3.6. Antiradical Activity against DPPH

The DPPH assay was performed following the methodology presented by Vignoli, Bassoli, and Benassi [3]. In brief, a 10 µL of sample solution (3 mg/mL) was mixed with 1 mL of 0.1 M acetate buffer (pH 5.5), 1 mL of ethanol, and 0.5 mL of 250 µM ethanolic DPPH solution. The control solution was prepared without using the coffee solution. The blank solution was prepared as above, except or the DPPH solution. The absorbance was measured with a Nanocolor UV/VIS II spectrophotometer at 517 nm and after 10 min of solution preparation. The inhibition ratio was calculated with the use of the following equation [41]:

Inhibition ratio (%) = ((Ac − As)/Ac) × 100

where

Ac—absorbance of a control
As—absorbance of a sample

The IC50 was determined using regression model following the procedure of Shimamura et al. [41].

### 3.7. Statistical Analysis

The data from the analysis, that was performed in triplicate, underwent statistical evaluation. The comparison of quantitative variables in the four groups was performed using the Kruskal–Wallis test. After detecting statistically significant differences, post hoc analysis was performed using the Dunn’s test to identify statistically significant groups. The analysis adopted a significance level of 0.05 and was performed in R software, version 4.1.0 [42].

## 4. Conclusions

The study results show that the choice of the method of coffee cherry processing to some extent affects the functional properties of the coffee beverage. Natural and anaerobic methods allow the highest caffeine concentration to be retained, indicating that coffee beverages obtained from beans subjected to those processing methods are characterized by better properties of central nervous system stimulation. Nevertheless, the selection of processing method does not have a significant influence on the total phenolic content and antiradical activity of coffee. However, it may still affect the phenolic profile of the coffee, and further research in that respect therefore needs to done.

The identified differences concerning water content and pH, along with the lack of significant discrepancies in titratable acidity, may correspond to the influence on the taste and aroma of coffee. Therefore, further research concerning the influence of the indicated processing methods on the sensory profile of coffee is needed.

The research results add new data to knowledge about the influence of coffee fruit processing on the final functional characteristics of coffee. However, there are some limitations to this study. They include: specific place of coffee plant cultivation, which may affect the chemical composition of coffee fruits and fermentation microbiota; and processing conditions, which may vary slightly between different producers and time of the year. Therefore, further research is needed to confirm the outcomes of this study.

## Figures and Tables

**Table 1:** Selected functional and quality parameters of tested coffee samples representing different post-harvest (pre-roasting) processing methods.

Parameter		Sample (*n* = 3)				*p*
		Washed–Extended Fermentation—A	Full Washed—B	Natural—C	Anaerobic—D	
Water (g/100 g)	mean ± SD	2.46 ± 0.02	2.68 ± 0.03	2.24 ± 0.03	2.63 ± 0.02	*p* = 0.019 * B,D > C
pH	mean ± SD	5.08 ± 0.03	5.04 ± 0.02	5.04 ± 0.01	4.98 ± 0.02	*p* = 0.019 * A > D
Titratable acidity (mol L^−1^ NaOH per 100 g)	mean ± SD	18.5 ± 0.5	18.83 ± 0.76	18.83 ± 0.76	18.83 ± 0.29	*p* = 0.811
Caffeine (g/100 g)	mean ± SD	1.672 ± 0.010	1.666 ± 0.009	1.758 ± 0.008	1.758 ± 0.014	*p* = 0.04 * D > A,B,C > B
TPC (mg GAE/g)	mean ± SD	38.81 ± 1.88	37.51 ± 0.78	37.93 ± 2.21	40.12 ± 1.81	*p* = 0.273
DPPH IC₅₀ (μg mL^–1^)	mean ± SD	21.59 ± 1.8	19.37 ± 0.18	20.16 ± 2.57	23.54 ± 1.3	*p* = 0.129

*—indicates statistical significance.

## Data Availability

The data presented in this study are available on request from the corresponding author.
